# Dr. Dilip Mahalanabis (1934-2022): Trailblazer in Diarrheal Disease Management

**DOI:** 10.7759/cureus.62241

**Published:** 2024-06-12

**Authors:** Umesh Kawalkar, Amar Mankar, Priti Kogade, Dinesh Naitam

**Affiliations:** 1 Community Medicine, Government Medical College (GMC) Akola, Akola, IND; 2 Community Medicine, Datta Meghe Institute of Higher Education and Research, Wardha, IND; 3 Public Health and Maternal and Child Health, National Tobacco Control Program, Public Health Department Akola, Akola, IND; 4 Dentistry, Government Medical College (GMC) Akola, Akola, IND

**Keywords:** cholera outbreak, oral rehydration solution, pediatrics emergency, public health and social work, historical vignette

## Abstract

Dilip Mahalanabis, an esteemed Indian pediatrician, revolutionized global health through his pioneering work in combatting diarrheal diseases, particularly during the Bangladesh War of Independence in 1971. His development of oral rehydration therapy (ORT) provided a simple, cost-effective solution that significantly reduced mortality rates among cholera patients. Mahalanabis' dedication to equitable healthcare, evidenced by his leadership roles in organizations such as the World Health Organization (WHO), underscores his legacy as a champion for vulnerable populations. ORT's widespread adoption has democratized treatment, empowering communities and drastically reducing mortality rates associated with diarrheal diseases.

## Introduction and background

**Figure 1:**
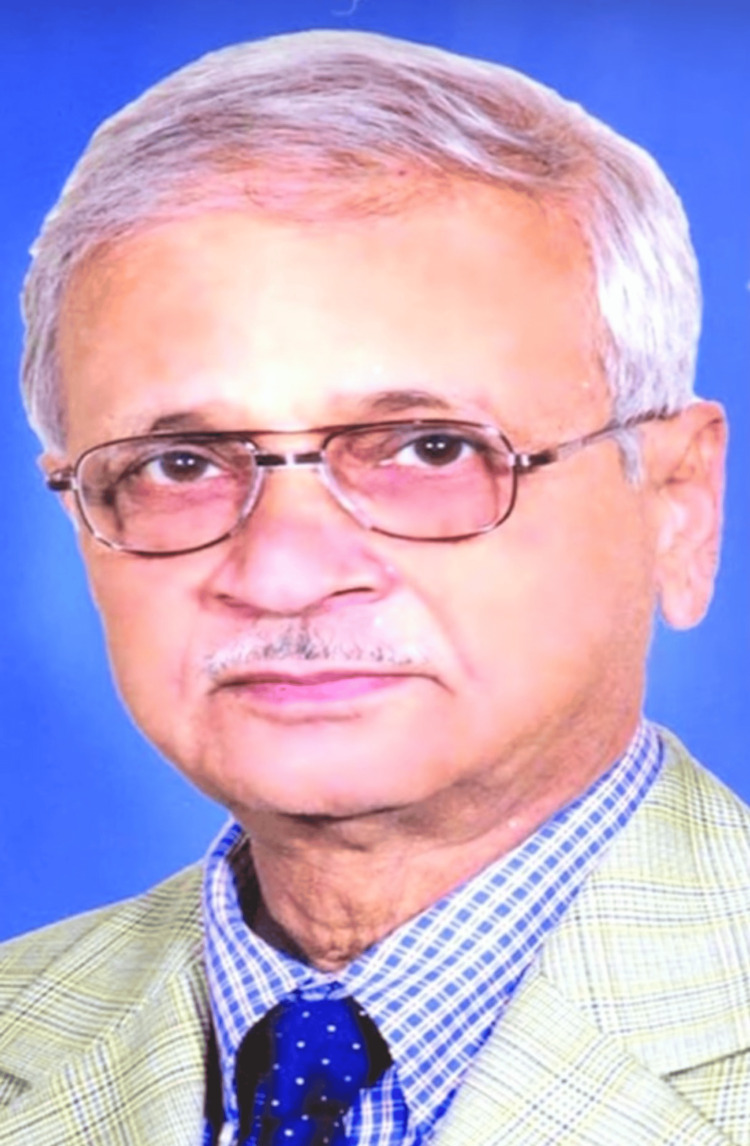
Dr. Dilip Mahalanabis (1934-2022) Under Creative Commons Attribution-Noncommercial-Share Alike 4.0 License. Source: The Wire Science

The main purpose of this article is to highlight the indisputable contribution of Dr. Dilip Mahalanabis (Figure 1), emphasizing his groundbreaking work in the control of diarrheal disease by oral rehydration therapy (ORT), potentially the most important medical advance of the 20th century [1]. Dilip Mahalanabis, an Indian pediatrician, occupies a revered place in the annals of global health for his pioneering contributions to combatting diarrheal diseases. His remarkable journey from his humble beginnings in British India to becoming a renowned figure in pediatric medicine is a testament to the transformative power of dedication and innovation in healthcare [1,2]. Mahalanabis' groundbreaking work in ORT during the tumultuous period of the Bangladesh War of Independence in 1971 fundamentally changed the landscape of diarrheal disease management. His tireless efforts, often amidst challenging circumstances, led to the development of a simple yet remarkably effective solution that saved countless lives [1]. Beyond his seminal research, Mahalanabis' commitment to equitable healthcare and his leadership in global health organizations such as the World Health Organization (WHO) and the International Centre for Diarrhoeal Disease Research (ICDDR,B) further solidify his legacy as a champion for the most vulnerable populations [1]. As we delve into the life and work of Dilip Mahalanabis, we uncover not just a story of medical innovation but a narrative of compassion, resilience, and the unwavering pursuit of a healthier world for all.

## Review

Mahalanabis' life and career

Dr. Dilip Mahalanabis, an esteemed Indian pediatrician, scientist, and public health specialist renowned globally for his work on oral rehydration solutions (ORSs), was born on November 12, 1934, in Kishoregunj (currently in Bangladesh), Mahalanabis received his medical degree from Calcutta Medical College and Hospital (Kolkata, India) in 1958. After graduation, he pursued postgraduate education in London, United Kingdom [1]. In the 1960s, Mahalanabis conducted seminal studies on ORT at the Johns Hopkins University International Center for Medical Research and Training in Kolkata, illuminating the efficacy of ORS in preventing and treating dehydration associated with diarrheal diseases. His groundbreaking work during the Independence War of Bangladesh in 1971 exemplified his visionary approach to public health, as he spearheaded efforts to combat cholera outbreaks by advocating for the widespread adoption of ORS. Mahalanabis' innovative use of a simple solution comprising salt, water, and sugar in refugee camps significantly reduced the case fatality rate from cholera, garnering international recognition for his epoch-making discovery [3]. Throughout his distinguished career, Mahalanabis' unwavering commitment to improving health outcomes transcended borders and disciplines. He played a pivotal role in shaping global health policies by collaborating with organizations like the WHO and the ICDDR,B. His advocacy for affordable and accessible healthcare earned him the admiration of colleagues and peers, who revered him as a humble yet influential champion for the poor and marginalized [3].

Mahalanabis' contributions to public health were recognized with numerous accolades, including the prestigious Pollin Prize in 2002 for his groundbreaking research on ORT and the Prince Mahidol Award, Thailand's highest civilian honor, in 2006. Despite his remarkable achievements, Mahalanabis remained committed to serving his community, exemplified by his philanthropic endeavors, such as establishing a pediatric ward in the Kolkata-based Institute of Child Health in memory of his late wife, Jayanti Mahalanabis. Throughout his career, Mahalanabis' legacy extended beyond scientific achievements to embody the values of empathy, humility, and dedication to improving the lives of others. His indelible impact on global health, particularly in diarrheal disease management, continues to inspire generations of researchers and healthcare professionals worldwide, cementing his status as a towering figure in public health and humanitarianism [2,3].

Mahalanabis' work on pioneering ORT

Cholera has been a significant health threat, as documented in Chinese, Greek, and Sanskrit literature, since ancient times. With its intricate network of waterways and swamps, the Ganges River Delta has been a historical hotspot for cholera since the 19th century. The world has experienced seven cholera pandemics, the first of which began in 1817 in the Jessore district of India (now in Bangladesh). The year 1854 was particularly devastating, with 23,000 cholera deaths reported in Great Britain alone [4]. This year also marked a pivotal moment in public health when English physician John Snow identified the link between contaminated water supplies and the spread of cholera. Another major advancement occurred in 1883 when German physician Robert Koch, a pioneer in bacteriology, isolated the *Vibrio cholerae* bacterium during his studies of outbreaks in Egypt and India. The seventh pandemic of cholera, caused by a new strain known as "El Tor," originated in Indonesia and reached Africa in 1970, where it remains a persistent problem. The risk of cholera outbreaks significantly increases during conflicts, natural disasters, and other crises that disrupt access to clean water and sanitation. Before the introduction of ORT, mortality due to diarrhea was alarmingly high, particularly among children under five years old. Estimates from the early 1980s indicated that diarrhea was responsible for approximately 4.6 million deaths annually among children under five globally. It was implicated in nearly a third of all deaths in this age group. This staggering mortality rate highlighted the urgent need for effective interventions to address diarrheal diseases and reduce the associated death toll [4,5,6].

In 1971, Bangaon, India, bore witness to a humanitarian crisis of staggering proportions as millions of refugees poured in from East Pakistan, now Bangladesh. As the monsoon arrived in June, bringing the feared cholera outbreak, Dr. Dilip Mahalanabis and his team in Bangaon faced a staggering influx of patients. As the number of refugees arriving at the camp steadily increased, the number of cases Mahalanabis and his team treated gradually became a major outbreak. By mid-June, the camp population had surged to approximately 350,000, with about 6,000 new cases daily. Despite their efforts, the limited availability of intravenous (IV) saline proved insufficient to meet the overwhelming demand, resulting in a devastatingly high mortality rate, particularly among children. Faced with the grim reality of losing the battle against cholera, Mahalanabis made the bold decision to implement ORT on a massive scale, even entrusting its administration to individuals without medical training. This unconventional approach marked a significant departure from traditional medical wisdom but proved a turning point in the fight against cholera. The medical community cautioned Dr. Dhiman Barua, the WHO cholera specialist, against allowing inexperienced staff to administer ORT. Barua had vivid memories of a cholera outbreak in 1932 in what is now Bangladesh, where he witnessed a devastating loss of life due to the lack of saline for treatment. Barua was determined to prevent a repeat of the 1932 tragedy, but the early 1970s presented significant challenges. The seventh cholera pandemic, which began in Indonesia in 1961, had spread widely, leading to numerous pleas for assistance from the WHO. Barua recalls a particularly desperate telegram from Africa: "I see children swimming in the cholera stools of their parents." The demand for IV saline was immense, but the cost of supplying it to the 40 countries affected by cholera far exceeded the WHO's budget [4].

Mahalanabis, recognizing the urgency of the situation, made a bold decision. He opted for a simple rehydration formula comprising sugar, salt, and bicarbonate of soda, ingredients known to be effective in combating severe cholera cases. Although potassium, an essential mineral, was absent due to its scarcity, Mahalanabis believed this straightforward mixture could still save lives. Setting up a makeshift production facility in the library at Johns Hopkins Center, Mahalanabis and his team meticulously measured and packed precise proportions of the ingredients into plastic bags, accompanied by instructions for dissolution in water. At the camp, Mahalanabis organized the staff into two groups: one attending to the severely ill with IV saline, while the other distributed the ORS to those able to drink. This marked the beginning of a large-scale experiment involving thousands of critically sick individuals. Mahalanabis faced challenges such as vomiting from excessive salt and reluctance among patients to consume the solution, but he persevered, closely monitoring for adverse effects and adjusting the approach as needed. Gradually, the treatment began to yield positive results, with a significant decrease in mortality rates observed over time. The success of the approach became evident as the number of deaths dwindled, and patient admissions declined. By the end of the outbreak, Mahalanabis and his team had treated thousands of individuals, with a remarkable reduction in mortality rates compared to traditional methods. ORT faced doubt and skepticism in treating diarrhea patients, but its clear success at Bangaon proved it could save lives during cholera outbreaks. [4,7].

ORT balanced solution comprising electrolytes (e.g., sodium, potassium, chloride) and glucose, aimed at augmenting the intestinal absorption of water and vital nutrients. Biochemically, ORT operates through the mechanism of osmosis. ORT works by using glucose to create an osmotic gradient that drives the absorption of sodium and water across intestinal cells, replenishing lost fluids and electrolytes [8]. It is noteworthy that Susruta, an eminent figure in Ayurvedic medicine during ancient times (circa 1000-500 BCE), advocated for the consumption of a solution consisting of tepid water infused with rock salt and molasses as a remedy for diarrhea. Additionally, historical records document sporadic instances of the beneficial use of oral glucose-saline solutions in treating cholera. However, these anecdotal accounts failed to gain widespread acceptance due to the absence of robust scientific validation and controlled studies [9].

The WHO, faced with the growing urgency of cholera outbreaks globally, eventually embraced ORT as a viable solution. The events in Bangaon in 1971 served as a catalyst for a paradigm shift in global attitudes toward ORT, marking a significant milestone in the fight against cholera and diarrheal diseases worldwide. The simplicity and efficacy of ORT, comprising a mixture of sugar, salt, and bicarbonate of soda, emerged as a beacon of hope amidst the crisis. Mahalanabis and his team persisted through initial challenges in ensuring proper administration and dosage, converting the Johns Hopkins Center into a makeshift factory to produce the solution. By empowering paramedics and family members to dispense ORT and closely monitoring patients for adverse reactions, Mahalanabis orchestrated a monumental effort to save lives [4].

The success of ORT in Bangaon not only demonstrated its effectiveness in treating cholera but also challenged entrenched skepticism within the medical community. Mahalanabis' approach, initially met with resistance, showcased remarkable outcomes acknowledged by the WHO's cholera specialist, Dr. Dhiman Barua, marking a global shift in managing diarrheal diseases through widespread ORT adoption. Barua's subsequent clinical trials in the Philippines further validated the efficacy and safety of ORT, paving the way for its widespread adoption as a lifesaving intervention worldwide. This breakthrough laid the foundation for the WHO's Diarrheal Diseases Control program, launched in 1978 with a focus on children under five. The WHO issued guidelines advocating ORS for all types of diarrhea, alongside continued feeding and limited antibiotic use. Collaborating with UNICEF and aid agencies, WHO supported the development of national diarrheal disease control programs, leading to widespread adoption and local production of ORS [4,8].

Continued research and innovation enhanced ORS effectiveness, with adaptations like substituting sodium citrate for sodium bicarbonate improving stability and affordability. Zinc supplementation alongside ORS showed promising results in reducing diarrheal episodes and complications. Monitoring and evaluation mechanisms demonstrated the impact of ORS, contributing to a substantial decline in diarrheal deaths among children globally [10]. One of the reasons the international medical journal The Lancet once described it as “potentially the most important medical advance of the 20th century” [11]. Agnimita Giri Sarkar from the Institute of Child Health in Kolkata, India, remarked to The Lancet Infectious Diseases that the contributions of Dr. Mahalanabis were instrumental in saving approximately 54 million lives, especially among children, over the course of three decades [3]. However, challenges persist due to inadequate sanitation and hygiene, which remain fundamental factors in diarrheal disease transmission. Efforts to improve water supply and sanitation continue, underscoring the ongoing importance of ORS as a lifesaving intervention in combating diarrheal diseases.

## Conclusions

Dr. Dilip Mahalanabis' revolutionary work in the field of public health, particularly his research on ORT, was born out of necessity during a devastating cholera outbreak in Bangaon, India, in 1971. ORT revolutionized the treatment of dehydration associated with diarrheal diseases, offering a simple yet effective solution that has since saved countless lives worldwide. ORT is considered one of the most significant medical breakthroughs of the 20th century, offering hope and healing to communities facing the scourge of diarrheal diseases. As we reflect on Mahalanabis' remarkable legacy, we are reminded of the power of human ingenuity and compassion in confronting the greatest health challenges of our time, inspiring future generations to continue the fight for health equity and dignity for all.
